# Gastrointestinal and metabolic effects of noodles-based konjac glucomannan in rats

**DOI:** 10.29219/fnr.v63.1997

**Published:** 2019-12-13

**Authors:** Yun Zhou, Jiangdan Qin, Yongquan Wang, Yichen Wang, Yongqiang Cheng

**Affiliations:** 1 Beijing Advanced Innovation Center for Food Nutrition and Human Health, College of Food Science and Nutritional Engineering, China Agricultural University, Beijing, China; 2 College of Food Science, Southwest University, Chongqing, People’s Republic of China

**Keywords:** konjac glucomannan, food matrix, noodles-based, digestion, glycemic response, satiety

## Abstract

This study was conducted to investigate the hypothesis that the beneficial metabolic effects of dietary fiber, konjac glucomannan (KGM), related with *in vivo* digestion might be altered if the complicated food matrix was taken into consideration. A diet of precooked noodles (PN), as widely produced and consumed in Asia, was used to simulate an actual food context. Assays were conducted with male Wistar rats (*n* = 80); the rats were divided into five groups and fed with either PN (control), PN supplemented with medium-dose KGM (MK), precooked low-dose KGM-supplemented noodles (LKD), precooked medium-dose KGM-supplemented noodles (MKD) or precooked high-dose KGM supplemented noodles (HKD). The time-dependent changes in blood glucose and the sensitivity to insulin after intragastric administration were determined to evaluate the postprandial glycemic response. The activity of intestinal Na^+^-K^+^-ATPase and the levels of gut hormones including motilin, cholecystokin, GLP-1, and orexin were also determined to provide insights into the function of gastrointestinal motion and after-meal hormonal feedback in each group. The noodles-based KGM showed much more efficacy in sustaining glucose homeostasis compared with KGM supplemented in a diet of noodles, indicating there might be potential long-term health outcomes of satiety and energy balance using noodles-based KGM. The postprandial glycemia was largely moderated by LKD and MKD. Despite the significant reduction in the production of glucose, MKD caused insensitivity to insulin–blood glucose regulation and a rapid gut negative feedback following a severe blood glucose fluctuation. In conclusion, the health-promoting benefits of KGM supplements on glycemic response highly depend on the type of matrix and the dose of KGM.

## Popular scientific summary

- Konjac glucomannan is a soluble dietary fiber, which has been proven to be effective in lowering postprandial glycemic response after intake as a food supplement.
- This study investigated whether konjac glucomannan in a noodles-based food matrix has a beneficial effect on metabolism related with digestion.
- KGM inhibited in vitro enzymatic hydrolysis of wheat starch and wheat gluten nearly dose-dependently.
- KGM slowed the rise of postprandial blood glucose and increased satiety by affecting intestinal metabolism of rats.

The glycemic index (GI) is associated with the postprandial blood glucose response of ingestion of a food with available carbohydrates. Intake of refined carbohydrates will raise postprandial blood glucose rapidly and even presents a risk factor associated with diseases including diabetes. Commonly eaten white noodles are readily digestible and their GI is really high (70 and above), whereas low-GI foods can control the release of glycosylated hemoglobin and fructosamine which lead to more stable postprandial blood glucose (1).

Dietary fiber is a class of ubiquitous polysaccharides that are widely distributed in food. Soluble dietary fibers appear to enhance the viscosity of digestion, decrease the gastric emptying rate, and affect propulsion caused by small intestine peristalsis (2). Muffins with high content of glucan and resistant starch in the body can reduce postprandial blood glucose and insulin levels (3). However, there is a growing interest in the performance of dietary fibers in the actual food context regarding *in vivo* postprandial blood glucose response. Accumulating evidence suggested that certain food contexts, through a variety of mechanisms, might alter the pattern of intestinal glucose uptake. The digestibility of dietary fiber extruded rice starch is remarkably lower than that of natural rice, and the GI decreases when the dietary fiber content is >6% (4). Dietary fiber presented in ‘whole food’ could destroy the mucin network structure on the intestinal mucosa and inhibit the transmembrane transport of nutrients (5). Despite substantial physiological function improvement in taking dietary fibers, it could lead to liver cancer in mice with intestinal microbial imbalance (6).

Konjac glucomannnan is a water-soluble, electrically neutral polysaccharide obtained from the konjac tuber. At molecular level, it is polymerized by d-mannose, and d-glucose with the side chain C-6 has an α-1, 4- glycoside bond and a small amount of acetyl (7). Physiological functions of KGM have been reported, including reducing blood glucose (8), lowering serum cholesterol (9), promoting defecation (10), and losing weight (11). Most of these effects can be attributed to the high viscosity of KGM, which prolong digestion time and slow the rate of absorption of nutrients from the small intestine, resulting in stabilized low postprandial blood glucose levels (12). Since viscous fibers such as KGM are contained in the diet, satiety can be promoted by regulating the release of these gastrointestinal hormones. The α-1,4-pyranoside bond on the KGM side chain can only be hydrolyzed by α-mannase at the end of the small intestine and in the colon (13, 14), where KGM can be decomposed by microbial fermentation in the colon, regulating the synthesis of short-chain fatty acids such as acetic acid and propionic acid; reducing the endogenous synthesis of cholesterol, fatty acids, and LDL; and producing hypolipidemic effects (15). Related research have proved that fermentable dietary fiber promotes the secretion of glucagon-like peptide-1 (GLP-1) more efficiently than non-fermentable fiber (16). Some soluble dietary fibers, such as inulin, can also be fermented in the colon and have been shown to alter the levels of peptide YY (PYY) and ghrelin in overweight and obese adults (17). Incorporation of KGM, xanthan gum, or bacterial cellulose in various plant-derived starches could synergistically prevent enzymes from contacting substrates and affect carbohydrate and lipid metabolism, suppressing insulin resistance, thereby preventing the development of diabetes and cardiovascular diseases (18).

This work was aimed to evaluate how differences in KGM impact glucose uptake and gastrointestinal hormone *in vivo* and to assess whether the effect of KGM foods on digestive regulation is comparable to that of KGM foods as dietary supplements to understand the differences in molecular interactions during food processing.

## Materials and methods

### Materials

Konjac glucomannan (KGM) was kindly provided by Shiyan Huaxianzi Konjac Products Co., Ltd. (Hubei, China). The contents of moisture, ash, glucomannan, crude protein, and fat were 3.7 g/100 g, 0.8 g/100 g, 87.8 g/100 g, 0.6 g/100 g, and 0.1 g/100 g, respectively. Wheat flour (Kerry Oil & Grains Industrial Co. Ltd., China) was used throughout the experiment, and the contents of moisture, ash, crude protein, starch, fat, and total dietary fiber were 12.83 g/100 g, 0.89 g/100 g, 11.2 g/100 g, 73.5 g/100 g, 1.6 g/100 g, and 1.21 g/100 g, respectively. The contents of moisture, ash, crude protein, amylose, and amylopectin in starch were 11.5 g/100 g, 0.89 g/100 g, <0.3 g/100 g, 17.78 g/100 g, and 66.1 g/100 g, respectively. The contents of moisture, ash, and protein in gluten were 6.34 g/100 g, 0.89 g/100 g, and 87.2 g/100 g respectively. Glucose determination kit (Ld 60201) was purchased from Laibang Biotechnology Co., Ltd (Beijing, China). Iodine (^125^I) insulin radioimmunoassay kit (KJEIA0041D), motilin radioimmunoassay kit (KDEIA0087), and cholecystokinin radioimmunoassay kit (KDEIA0091) were purchased from Kangjia Hongyuan Biotechnology Co., Ltd (Beijing, China). Ultramicro assay kit for Na^+^-K^+^-ATPase (A016-1) was purchased from Nanjing Jiancheng Biology Engineering Institute (Nanjing, China). Ghrelin ELISA kit (EK-031-31), glucagon-like peptide-1 ELISA kit (EK-028-11), and orexin A radioimmunoassay kit (EKE-003-30) were purchased from Phoenix Pharmaceuticals Inc. (USA). All the other chemicals and reagents were of analytical grade.

### In vitro digestion

KGM of 1 wt%, 3 wt%, and 5 wt% was thoroughly mixed with wheat starch and gluten. The mixed powder was slowly dispersed in distilled water and continuously stirred for 30 min to prepare 20 wt% suspension. The suspensions were heated at 95°C for 5 min and then cooled to room temperature. The boiled suspensions were freeze-dried, ground, and sieved for further use. The digestibility of starch was determined according to the method of Singh et al. (19). The digestibility of gluten was determined according to the method of Wang et al. (20).

### Noodles preparation

Noodles were prepared according to the method described by Zhou et al. (21) with some modifications. KGM was thoroughly mixed with wheat flour by weight ratios of 2%, 4%, and 8%. The KGM–wheat flour blends (200 g) were blended with 50–60% water in a small-scale kneader (Kenwood, UK) using planetary kneading at a low speed for 8 min over a 30-s interval every 4 min. The noodles were allowed to rest for 10 min and sheeted on a noodle machine (Kenwood, UK) with an initial gap of 3.0 mm followed by progressively narrowing gaps to 1.2 mm to ensure homogeneity. Noodles of strands of 3 mm in width and 20 cm in length were produced by cutting blades (Kenwood, UK). The noodles were boiled in boiling water for 4 min and allowed to cool to room temperature. The precooked noodles were freeze-dried, grounded, and sieved blank to obtain blank precooked noodles (control), precooked noodles based 2 wt% KGM (LKD), precooked noodles based 4 wt% KGM (MKD), and precooked noodles based 8 wt% KGM (HKD). Precooked noodles supplemented with medium-dose KGM (MK) were prepared by adding 4 wt% KGM to the blank precooked noodles.

### Laboratory animals and sample collection

The male Sprague Dawley rats (average weight, 300 g) were purchased from Huafukang Biotech (Beijing, China) and raised in a specific pathogen-free (SPF) environment with free access to feed and water. After a week of adjustable feeding, 80 rats were randomly divided into five groups: Control, MK, LKD, MKD, and HKD. Rats were fasted for 12 h before orbital sinus blood sampling, and these samples were immediately freezed. Treated noodles containing different dose of KGM were suspended into water (0.1 g/mL), and the rats received 2 mL of the suspensions per 100 g of weight by gavage. After 15, 30, 60, and 120 min of gavage treatment, 4 rats were randomly selected and their blood samples were taken by puncturing the orbital sinus while rats were under diethyl-ether anesthesia. Anticoagulant (disodium hydrochloride solution) was added to the plasma, and the rest of the plasma was placed at −80°C for isolating serum.

#### Blood glucose content measurement

The blood glucose was determined by glucose determination kit; 10 μL of serum was mixed with 1 mL of R1 reagent. The absorbance at 505 nm was recorded to calculate the blood glucose content.

#### Insulin level measurement

Insulin level was determined using iodine (^125^I) insulin radioimmunoassay kit; 200 μL of serum was mixed with the same volume of marker and antibody. The mixture was incubated at 4°C for 18 h; 1,000 μL of separating reagent was added and thoroughly mixed, followed by incubation in 4°C for 15 min and centrifugation at 3,600 rpm for 20 min. The supernatant was abandoned, and the cpm value of the sediment was measured to calculate the insulin level.

### Animal physiological indicators determination

#### Motilin concentration

Motilin was measured using the radioimmunoassay kit; 100 μL of plasma taken from executed rats after 30 min of gavage treatment was thoroughly mixed with 100 μL of antiserum. After incubation at 4°C for 24 h, 100 μL of ^125^I–MTL was added and thoroughly mixed, followed by incubation at 4°C for 24 h. Then, 500 μL of separating reagent was added and placed at room temperature for 20 min, followed by centrifugation at 3,500 rpm for 25 min at 4°C. The supernatant was abandoned, and the cpm value of the sediment was measured to calculate the motilin concentration.

#### Na^+^-K^+^-ATPase activity

The plasma collected from executed rats after 60 min of gavage treatment was centrifuged at 50,000 rpm for 10 min, and the serum in the supernatant was obtained for Na^+^-K^+^-ATPase activity measurement by ultramicro assay kit for Na^+^-K^+^-ATPase; 0.1 mL of serum was thoroughly mixed with 0.1 mL of distilled water, 0.06 mL of reagent ten, and 0.42 mL of matrix liquid. The mixtures were incubated at 37°C for 10 min and mixed with reagent four, followed by centrifugation at 4,000 rpm for 10 min; 0.3 mL of the supernatant was collected and mixed with 1.0 mL of reagent five, reacting for 2 min. After adding 1.0 mL of reagent six and standing for 5 min, absorbance at 636 nm was recorded to calculate the enzyme activity.

#### Cholecystokinin concentration

Cholecystokinin (CCK) was determined by radioimmunoassay kit; 300 μL of plasma was thoroughly mixed with 100 μL of antiserum. After incubation at 4°C for 24 h, 100 μL of ^125^I –CCK was added and thoroughly mixed, followed by incubation at 4°C for 24 h. Then, 500 μL of separating reagent was added and placed at room temperature for 45 min, followed by centrifugation at 3,500 rpm for 20 min at 4°C. The supernatant was abandoned, and the cpm value of the sediment was measured to calculate CCK concentration.

#### Ghrelin concentration

Ghrelin concentration was determined according to the Protocol for Catalog # EK-031-31 Ghrelin (Rat, Mouse) EIA Kit (Phoenix Pharmaceuticals, Inc) with no modification.

#### Glucagon-like peptide-1 concentration

GLP-1 concentration was determined according to the Protocol for Catalog # EK-028-11 GLP-1 (7-36)-Amide (Human, Rat, Mouse) EIA Kit (Phoenix Pharmaceuticals, Inc.) with no modification.

#### Orexin A concentration

Orexin A was determined according to the Orexin A (Human, Rat, Mouse) Extraction Free EIA Kit Protocol (Phoenix Pharmaceuticals, Inc.) with no modification.

### Statistical analysis

The results were statistically analyzed using SPSS (SPSS Inc., Chicago, USA). Analysis of variance (ANOVA) was used to determine significant differences between the results, and Duncan’s test was used to compare the means with a significance difference at the level of 0.05.

## Results and discussion

### Effect of KGM on in vitro digestibility of wheat starch

**Fig. 1:**
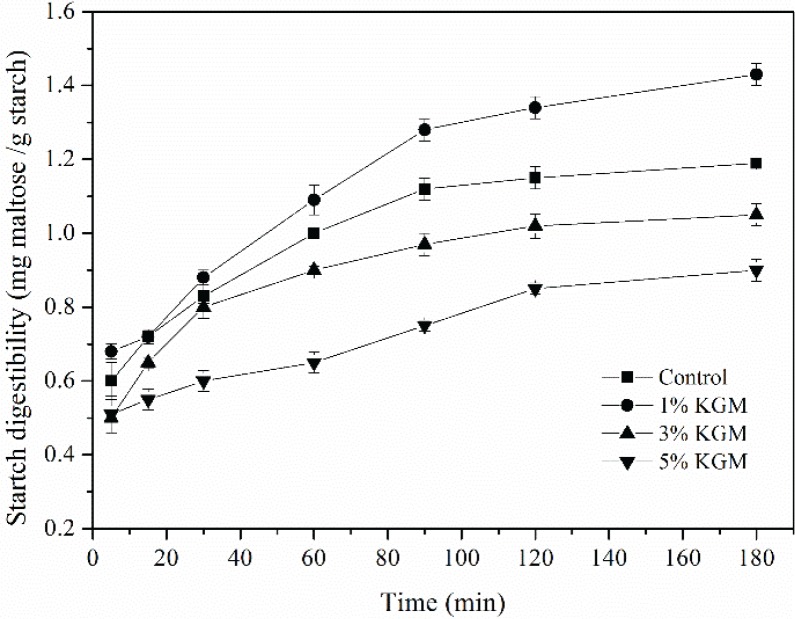
Digestibility of precooked wheat starch substituted with KGM of different concentrations (1 wt%, 3 wt%, and 5 wt%).

The digestibility of wheat starch with different contents of KGM was depicted in Fig. 1. The digestion was initially fast from 5 to 90 min. After 90 min of rapid digestion, the rates were alleviated. As shown in Fig. 1, the rate of digestion of gelatinized starch significantly decreased with 3% KGM and 5% KGM. Nevertheless, the *in vitro* digestibility of 1% KGM was significantly higher than those of the other samples. The differences in the inhibitory capacity on starch digestibility proved that the concentration of KGM is important for enzymatic hydrolysis, which could be explained in two aspects: firstly, in such a heterogeneous system, KGM molecules tended to be attached to the surface of starch particles, forming a barrier-like layer on the periphery, which blocked the contacts between enzymes and starch, especially the binding sites of enzymes and starch molecules. Besides, as the proportion of KGM increases, the thickening effects of KGM might limit mass transfer and further inhibit starch swelling (22–25); secondly, the thickening effects of KGM could also lower the rate of starch retrogradation by inhibiting the rearrangement of the leaching starch molecules to form the semi-crystal structure (26). Therefore, when the steric hindrance of enzyme–starch binding is dominant, a lower hydrolysis rate could be expected, which is the case of 3 and 5% KGM groups. Otherwise, KGM could even make it easier for amylase to hydrolyze the amorphous region of starch and eventually achieve an even higher starch digestibility at such a low concentration of 1% KGM.

### Effect of KGM on in vitro digestibility of wheat gluten

**Fig. 2:**
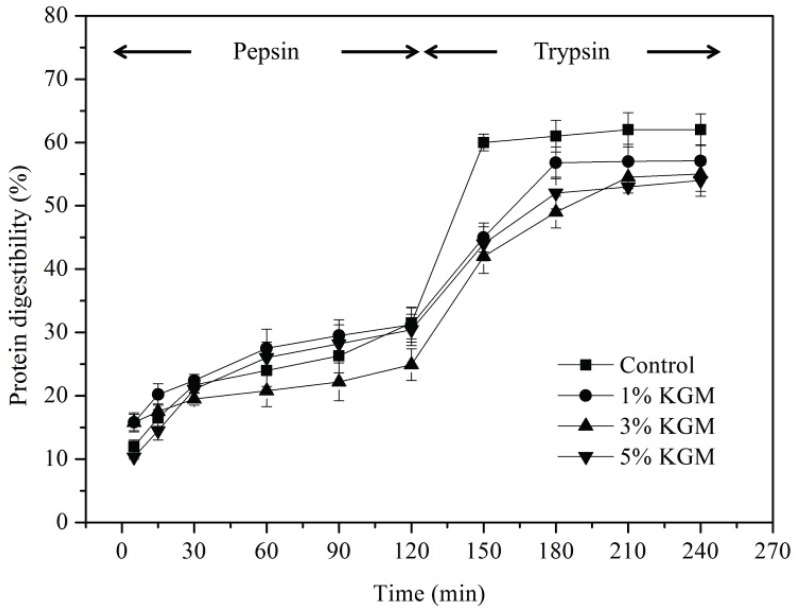
Digestibility of precooked wheat gluten substituted with KGM of different concentrations (1 wt%, 3 wt%, and 5 wt%).

The digestibility of wheat gluten with different contents of KGM was shown in Fig. 2. The rate of digestion was attenuated with time due to the decrease of substrates. Wheat gluten was hydrolyzed by pepsin at pH of 1.4 to simulate the digestion of protein in stomach and trypsin at pH 6.8 to simulate the digestion of protein in small intestine, of which the enzymolysis time is 120 min for each period.

It could be clearly seen that the protein digestibility of 3% KGM group increased by only 57.6% in 120 min, which was significantly lower than that of the other three groups (*P* < 0.05). The enzymolysis of control began to stabilize after 150 min. However, the enzymolysis of 1% KGM and 5% KGM groups was not complete until 180 min, while the enzymolysis of 3% KGM group was still in progress. There is no significant difference in the digestibility of protein for either control or KGM groups before 120 min. However, the rate of wheat gluten digestion in the control group was strongly up-regulated at the stage of trypsin enzymolysis. To some extent, the degradation of gluten protein by trypsin leads to the appearance of pores, partial network destruction, and the increase in pore size, which leads to the increase in digestion rate when entering the digestion stage of trypsin. A highly entangled network structure of indigestible KGM could be formed around the gluten (27). KGM could maintain a relatively stable status throughout the digestion process and interact with wheat gluten to shield protease active sites of proteins, which down-regulated the digestibility of wheat gluten in the whole process of enzymolysis. It is interesting to observe that the gluten protein digestibility of the 5% KGM group was higher than that of the 3% KGM group throughout the enzymolysis process. It was proposed that KGM is involved in the interaction with gluten by forming strong intermolecular hydrogen bonding system and competes with gluten for free water as the KGM concentration increased, resulting in incorrect folding of gluten protein and transforming compact structured protein into loose ones (28). Heating intensifies the denaturation of protein, disrupting the higher levels of protein structure and enabling a higher degree of enzymolysis of 5% KGM.

### Effect of noodles-based KGM on postprandial blood glucose

**Fig. 3:**
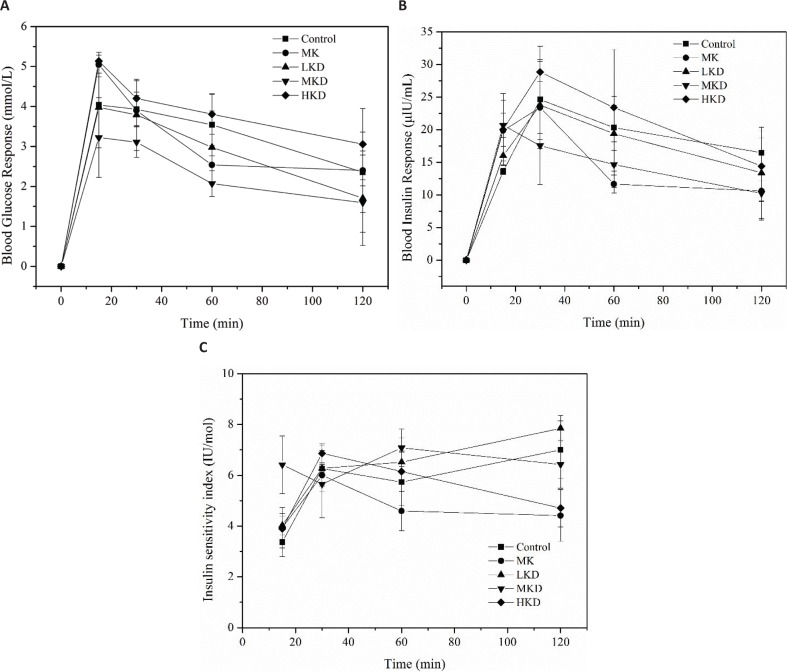
Effect of different noodles-based KGM on postprandial blood glucose: (a) changes in blood glucose response, (b) blood insulin response, and (c) insulin sensitivity of mice after intragastric administration. The animal was raised under normal experimental conditions, and it was free to feed and drink for 1 week. Data are representative of data obtained from four animals randomly selected from each group. LKD: precooked noodles based 2 wt% KGM, MKD: precooked noodles based 4 wt% KGM, HKD: precooked noodles based 8 wt% KGM, and MK: 4 wt% KGM mixed directly with precooked noodles. The error bars indicate the standard deviations of duplicate measurements.

The secretion of insulin is closely related to the level of blood glucose response, and both of them can be used to indicate the extent of digestion and absorption of food. As shown in Fig. 3a, the postprandial blood glucose levels of five groups were within the normal range. Rats of the MKD group showed a typical controlled glycemic process within 120 min after meal, which was significantly lower than the control group. KGM down-regulated the postprandial blood glucose level due to the slowing down of starch digestion, which was consistent with our observation of *in vitro* digestibility of wheat starch. However, the glucose level of the HKD group was even higher than the control group. The postprandial blood glucose response of MK group fluctuated dramatically. To be specific, the postprandial blood glucose level of MK group at 15 and 30 min was remarkably higher than that of the MKD group (*P* < 0.05), but there was no significant difference between these two groups at 60 and 120 min after feeding, suggesting how KGM was incorporated in a diet might be also important for functioning.

Fluctuations in blood glucose level impact the secretion of insulin, and the blood glucose level is regulated by insulin in turn. Insulin is the only known hormone in the body that lowers the blood glucose level. Figure 3b showed that the insulin level increased rapidly, which is similar to the glycemic response curve with a comparative delay. Except the MKD group, the insulin levels of the other four groups jumped at 30 min after feeding and decreased gradually afterwards. Meanwhile, the levels of insulin were all within the normal limit. The insulin levels of the rats in the 5 groups were similar at 2 h. The insulin response of MK group fluctuated sharply from 15 min to 1 h after intragastric administration and decreased rapidly after 1 h. After that, the insulin level was comparatively stable.

The sensitivity of the body to insulin is another important factor in stabilizing postprandial blood glucose. Figure 3c showed the insulin sensitivity (IS) index varying with time for different groups. After 15 min of feeding, the IS index of MKD peaked for the first time before the other groups which can be explained that the rapid growth of insulin is caused by the MKD group in Fig. 3b. The IS index of KD group was higher than that of the control group before 60 min, indicating that the KD group had increased the IS index to some extent, which was in accordance with previous studies (29). The IS index followed the order: LKD > Control > MKD > HKD > MK, when it is 2 h after feeding.

Our study revealed that the blood glucose of MKD was lower than that of MK, considering that MK was made by directly blending the medium-dose KGM with precooked noodles, which was different from the way of MKD making. The interaction of KGM and starch in the noodles-making process reduces the degree of starch gelatinization (30). The crystal structure of the A-type starch aggregate can be obtained in a larger amount, which can increase the content of slow-digested starch (SDS) in the food matrix (31). This suggested that KGM can not only affect the digestibility but also affect the interaction with other food components, such as starch. Although our *in vitro* experiments have shown that in a KGM-thickened system, starch enzymolysis can be reduced, the efficacy of soluble dietary fibers to attenuate the blood glucose level in complex mixing conditions such as the small intestine may not be simply attributed to changes in intestinal digestive viscosity (32). The time length of staying for foods in stomach and then transferring from stomach to small intestine should also be considered while predicting the absorption rate of blood glucose. Additionally, the transport rate in small intestine is also a significant factor for the blood glucose concentration because it determines the substrate concentrations for digestive enzymes in small intestine. However, previous study suggested that neutral dietary fiber polysaccharides such as arabinoxylan and β-glucan can hinder the transport of nutrients across the mucosa by disrupting the mucin network. Therefore, it is speculated that the electrically neutral KGM may have the same mechanism affecting the absorption of nutrients such as glucose in the intestine (33).

However, HKD group increased the blood glucose, which has seemingly opposite results with *in vitro* digestion. Protein gluten structure is a natural barrier to starch digestion. Premature hydrolysis of proteins or addition of a reducing agent that prevents the formation of disulfide bonds can increase starch digestibility and digestion rate (34). When KGM interacts with gluten, the correlation results indicate that KGM has a role in hindering the formation of gluten networks. Therefore, when considering the effect of KGM on food digestion and absorption efficiency, this factor needs to be taken into account. This explains that the blood glucose response of the HKD group is the highest among all groups (27, 28).

Blood glucose homeostasis is related with gastric emptying, glucose absorption, and transportation rate, which cannot be interpreted by *in vitro* experiments. The effects of KGM on regulating the digestion rate of starch *in vivo* can be speculated from the animal experimental results. However, whether KGM could also function on the gastrointestinal motility, emptying, and transport processes regarding maintaining a steady rise in postprandial blood glucose is still unknown.

### Effect of noodles-based KGM on gastrointestinal function

**Fig. 4:**
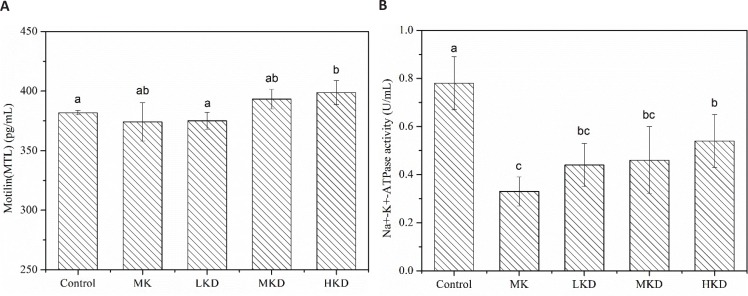
Concentration of plasma motilin (a) and the activity of serum Na^+^-K^+^-ATPase (b) of mice after intragastric administration. The samples of motilin and Na^+^-K^+^-ATPase were taken from the plasma of rats sacrificed at 30 and 60 min after gavage, respectively. Data are representative of data obtained from four animals randomly selected from each group. LKD: precooked noodles based 2 wt% KGM, MKD: precooked noodles based 4 wt% KGM, HKD: precooked noodles based 8 wt% KGM, and MK: 4 wt% KGM mixed directly with precooked noodles. Values are means ± SD. Different letters superscripted on the columns indicate significant difference, *P* < 0.05.

Motilin is periodically released during digestion process. Motilin receptors were found throughout the whole digestive tract. A higher level of motilin after diet could reduce food intake because of its inhibitory effect on gastric regulation (35). Significantly higher concentration of motilin was found in the HKD group compared with the other groups in Fig. 4a, which indicates that the intestinal tract movement needs to be improved to shorten the retention time of food debris in the intestinal cavity of HKD group rats.

Na^+^-K^+^-ATPase activity could be used to interpret the absorptive capacity of nutrients in the small intestine. When the Na^+^-K^+^-ATPase activity was decreased, the potential energy reserve was reduced as well, and as a result, the absorption of glucose, amino acids, and other substances in the intestinal tract was inhibited. As shown in Fig. 4b, intake of KGM could significantly lower the activity of Na^+^-K^+^-ATPase, but there is no significant difference in the effect of noodles based different amounts of KGM. The minimum activity of Na^+^-K^+^-ATPase was achieved in MK group (*P* < 0.05), indicating that the type of matrix where KGM was incorporated is critical to regulate the activity of Na+-K+-ATPase. It was demonstrated that the reduction of nutrient absorption capacity of KGM is related to its matrix. The activity of Na^+^-K^+^-ATPase would be lowered to accommodate these changes in the amount of small molecule nutrients needed to be transferred. Partial degradation of KGM in colon could produce short-chain fatty acids, decrease intestinal pH, and promote the passive regulation of Na+-K+-ATPase (36).

### Effect of noodles-based KGM on gastrointestinal hormones

**Fig. 5:**
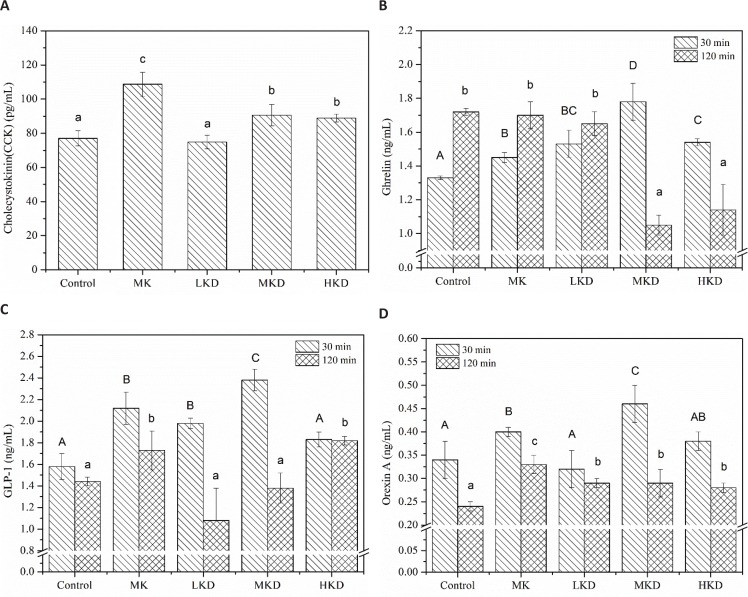
Effect of different noodles-based KGM on gastrointestinal hormones: (a) Concentration of in plasma cholecystokin, (b) plasma ghrelin, (c) plasma GLP-1, and (d) plasma orexin of mice after intragastric administration. Different striped columns represent plasma gastrointestinal hormone levels at 30 and 120 min, respectively. Data are representative of data obtained from four animals randomly selected from each group. LKD: precooked noodles based 2 wt% KGM, MKD: precooked noodles based 4 wt% KGM, HKD: precooked noodles based 8 wt% KGM, and MK: 4 wt% KGM mixed directly with precooked noodles. Values are means ± SD. Different uppercase and lowercase letters superscripted on the columns indicate significant difference, *P* < 0.05.

Cholecystokinin is the most extensively studied gastrointestinal satiety hormone secreted by the I-cells that line the intestinal tract. The main role of CCK is to promote the secretion of various digestive enzymes from the pancreatic acinus and enhance the release of GLP-1 to reduce food intake (37). In Fig. 5a, plasma CCK concentration was increased by KGM than that of the MK group which was significantly higher than the other groups. The secretion of CCK suggested that food was digested slowly and more time was required to complete the digestion. Previous studies showed that the release of CCK could be stimulated mainly by unhydrolyzed proteins and free fatty acids (38). Considering that the food system of MK was different from those of KD groups, changes in CCK concentration could be attributed to how the gluten was binded to KGM and the interactions between gluten and KGM which allows more intact gluten protein to promote the CCK secretion in the given food matrix. Additionally, a comparison *in vivo* study of dietary from different food matrixes at the same concentration showed that intake of dietary fibers coming from less-processed foods resulted in higher plasma CCK level, that is, β-glucan from oat bran was more capable than that from oat flour and oatmeal to enhance the CCK level (39).

Ghrelin, a gastrointestinal hormone synthesized and secreted primarily from the fundic mucosa of the stomach, appears to be an orexigenic signal which would increase in state of hunger or food deprivation. Figure 5b showed the concentrations of ghrelin in the plasma of rats from different groups at 30 and 120 min after feeding. The plasma ghrelin levels were significantly lower in the control group at 30 min after feeding, indicating that hunger of rats in this group was largely relieved. The maximum concentration of ghrelin appeared in MKD group, followed by the LKD and HKD groups. Ghrelin participates in stage II through the afferent nerve of the vagus nerve and coordinates the contraction of phase III with motilin (40). Combined with the results of Fig. 4a, it was further proved that rats from the HKD and MKD groups need to increase the hormone concentration to promote gastrointestinal motility. The ghrelin levels in the control, MK, and LKD groups were elevated at 120 min, while the hormone levels of the MKD and HKD groups were oppositely changed. The slow-reducing process of ghrelin secreted from gastric P/D1 cells at the later stage of digestion indicated that there is a delayed food digestion of rats from the MKD and HKD groups (41). Ghrelin is produced in the islet, which regulates blood glucose by limiting the release of insulin (42). Lower levels of ghrelin in the HKD group led to higher levels of insulin release, which was consistent with trend of postprandial blood glucose response. In addition, ghrelin also plays an important role in the regulation of long-term energy homeostasis. Previous studies have shown that ghrelin concentration in fasting plasma is negatively correlated with BMI value (43).

GLP-1 is a neuroendocrine polypeptide synthesized and secreted by enteroendocrine ‘L’ cells located within the ileum of the small intestine and colon. The biological activities of GLP-1 include stimulating the secretion and synthesis of insulin and enhancing insulin sensitivity, which are important for maintaining homeostasis in human body (44). Figure 5c shows the GLP-1 concentrations in plasma at 30 and 120 min after feeding. The GLP-1 concentration of MKD group was the highest, followed by the MK and LKD groups, of which GLP-1 concentrations were significantly higher than control. Among the three major nutrients, carbohydrates and lipids are most likely to stimulate precursor molecules to synthesize GLP-1 (45). The LKD, MK, and MKD groups produced more GLP-1 in the first 30 min possibly due to the relatively higher amounts of available carbohydrates. The final product of dietary fiber transformed from gut microorganism and specific enzyme repertoire is the short-chain fatty acid, which can affect the secretion of appetite hormone. It was speculated that KGM stimulates GLP-1 secretion by affecting lipid fermentation, resulting in a higher hormone level (46, 47). However, the relatively low level of GLP-1 in HKD group may be related to a high-viscosity environment, which impaired the effective stimulation of L-cells and concomitant GLP-1 release (48). The MK and HKD group maintained a comparatively stable GLP-1 level at 30 and 12 min. Although the concentration was lowered at 120 min, it was still significantly higher than the other groups. Previous studies have shown that slow digestion rate of raw corn starch can cause postponed secretion of GLP-1 (49). The minor variation of GLP-1 content in HKD group in the experiment suggested that the digestion of starch was slow, which kept blood glucose level at a long-term balanced state.

Orexin is a neuropeptide substance, which plays a physiological role in regulating appetite, glycolipid metabolism, and energy utilization through complex central and peripheral tissues (50). At 30 min after feeding, the orexin concentrations of rats from MK, MKD, and HKD groups were significantly higher than control (Fig. 5d), indicating the inhibitory effect of MK, MKD, and HKD on gastrointestinal motility, and the promotion of gastrointestinal movement and digestion of food in stomach was required. However, there is no dose–response relationship observed between the concentration of orexin and KGM. For the MKD group, rats exhibited the maximum concentration of orexin and the minimum blood glucose concentration. The negative correlation between orexin and blood glucose concentrations was demonstrated previously. At 120 min after feeding, orexin concentration was declined to around 0.3 mg/mL. The orexin concentrations of all KGM groups were significantly higher than that of the control, and MK group was the largest in orexin concentration. Fasting could increase the secretion of orexin along with the frequency of food intake and increased gastrointestinal peristalsis (51). Therefore, the increase in orexin secretion may be due to the regulation of energy compensation caused by the reduction in energy supply by food ingestion.

## Conclusion

*In vitro* enzymatic hydrolysis of wheat starch and wheat gluten was nearly dose-dependent on KGM concentration, but the interaction between KGM and two wheat components certainly interferes the hydrolysis probably by its thickening and steric hindrance effects. *In vivo* assays could reveal a correlation between blood glucose elevation, insulin secretion and gastrointestinal hormone release under such a precooked noodles-based KGM system besides the digestive enzymes-induced enzymatic reaction. This study indicated that the beneficial metabolic effects of KGM related with digestion were not similar within different food matrixes. There are wild fluctuations of glucose in the blood of MK. This can eventually lead to low insulin sensitivity and increased risk of obesity and diabetes. Even at the same dose of KGM, MKD was more desirable in quickly satisfying the appetite of rats, enhancing satiety and promoting the effect of insulin to maintain stable blood glucose. HKD group tended to maintain satiety at the time when the digestion process has been basically completed. In conclusion, metabolism is extremely complicated and varies with the type of food matrix. In the analysis of the impacts of KGM on digestion, it is necessary to consider the combined effects of food pattern on various indicators including enzymatic reaction, gastrointestinal motility, and hormones. Feeding of KGM incorporated in staple foods like noodles is better than the supplementation in reducing food intake and weight control.
